# Evolution of Focal Conic Domains in SmA-N Phase Transition

**DOI:** 10.3390/ma18030711

**Published:** 2025-02-06

**Authors:** Vincent Plée, Jordan Lacam, Gianni Pascoli, Claire Meyer

**Affiliations:** Laboratoire Physique des Systèmes Complexes, Université de Picardie Jules Verne, 33 rue Saint-Leu, 80039 Amiens, Francepascoli@u-picardie.fr (G.P.)

**Keywords:** liquid crystals, smectic A phase, nematic phase, phase transition, defects

## Abstract

Focal conics are nice geometric structures of liquid crystal phases which present periodicity such as smectic phase, cholesteric phase, etc. Here, we focus on focal conic domains (FCD) in smectic A liquid crystal. During a phase transition from smectic A to nematic, these FCDs evolve as the eccentricity increases until they completely disappear. Here, we present experimental observations of this phenomenon, along with a modeling approach that allows for an interestingly precise description of the phenomenon, revealing, in particular, a coefficient that seems to exhibit the behavior of a critical exponent.

## 1. Introduction

The observation of liquid crystals under an optical microscope is fascinating due to the presence of very nice defective textures. The nematic (N) phase, where the molecules are, on average, oriented along a direction, called the director, possesses disclination lines classified by Sir Ch. Frank [1]. The smectic A (SmA) phase shows different defects first discovered by G. Friedel and F. Grandjean [2]: the well-known focal conic domains [3,4] made either by ellipse and confocal hyperbola or by two confocal parabolas [5,6]. The Sm A phase also presents defects named simple and double helices, which were first discovered by C. Williams [7,8]; more recently the evolution of the double helices with temperature [9] or under the action of an electric field [10] has been reported. Whereas these phases are nowadays well-known, new discoveries have been recently reported, with special geometries in cholesteric [11] and in smectic phases [12].

On the other hand, a number of applications of FCDs have been proposed: for instance, FCDs as optical lenses [13], or for the elaboration of new types of smart windows. Polymer-stabilized liquid crystals have recently been used to maintain the focal conic domains in the nematic phase for smart window applications. The authors [14] have shown that UV polymerization of linear arrays of elliptic and hyperbolic FCD in the smectic A phase creates new functions of active privacy layers. This study has been extended to various defects (FCDs, stripe textures, double helices) of the twist–bend nematic phase [15]. An analysis based on hybrid anchoring in 8CB has been recently reported [16]. It enriches the knowledge of textures controlled by topological defects of liquid crystals. Their texture, called zigzag FCDs by the authors, is generated during a rapid cooling process. Furthermore, lithographic applications based on smectic liquid–crystalline materials [17] have been developed. The progress in these applications encourages us to revisit the smectic A/nematic phase transition, carefully observing textures upon very slowly increasing the temperature. In fact, many questions remain open. In particular, the question of the mechanism involved during the disappearance of focal conic domains, occurring typically less than one degree before the SmA-N phase transition, is not completely understood. Different mechanisms have already been presented [5,18].

**Figure 1:**
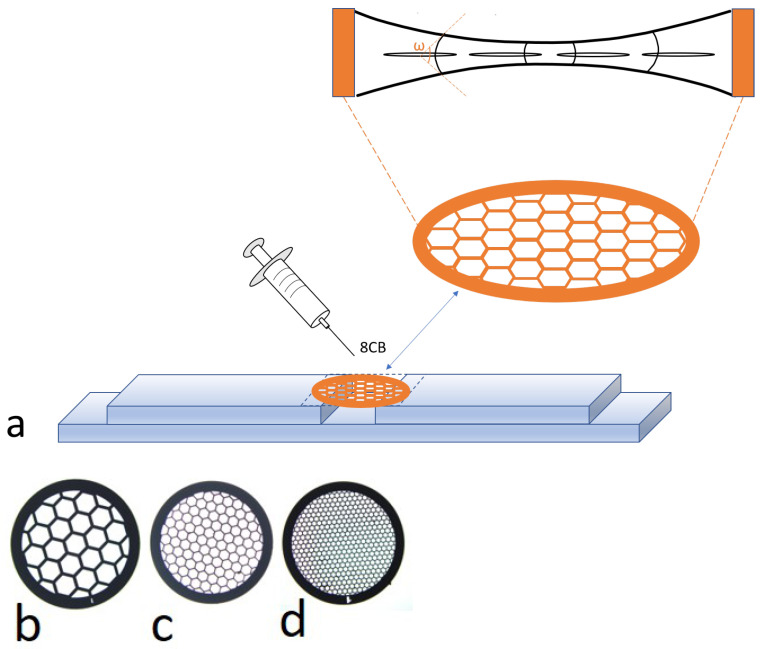
(**a**) Schematic representation of the experimental setup: a drop of 8CB has been deposited on a hexagonal grid. After annealing, the equilibrium shape of the film is shown in the upper part of the figure. The eccentricity of the ellipse is related to the dihedral angle ω by the relation $e\left(\omega \right)=\sin\frac{\omega }{2}$. (**b**–**d**) Top view of empty grids with numerous hexagonal holes, enabling repeated observation of the FCDs during temperature variation. Each grid measures 3.05 mm in diameter: (**b**) 50 mesh (G2405C); (**c**) 100 mesh (G2410C); (**d**) 200 mesh (G2450C).

In the literature, the presence of distorted, i.e., imperfect, FCDs has been reported [18], and the authors demonstrated that an imperfect FCD corresponds to a kinked FCD. A kink acts as an imperfection on a disclination: it results from the attachment of a dislocation to the disclination, leading to the formation of a kink. Changes in the number of dislocations attached to a disclination alter its shape, as illustrated in Figure 2b of [18]. Here, we do not focus on such imperfections; our FCDs are approximately treated as ideal. The seminal 1910 article by Friedel and Grandjean’s [2] addressed the issue of the evolution of the geometry of the FCDs: “The hyperbola bends as *e* increases; its asymptotes get closer. Finally, in the limit, when *e* reaches unity, the focal group reduces to a half-line, marked only in a small region”. This can be found on page 421. Following this line, our work focuses on this problem to precisely study the FCDs behavior very close to the transition towards the nematic phase, using experiments with very slow temperature variations. Here, we present special experiments using a device—usually used in TEM—made of a grid with a regular network of hexagonal holes of different sizes (Figure 1). This device (Figure 1) allows us to repeatedly check the SmA-N transition simultaneously in many holes (at low objective microscope magnification). Increasing the magnification permits us to focus on a single hole, while heating slowly allows us to carefully scrutinize the FCD evolution in a close neighborhood of the SmA-N phase transition.

## 2. Experimental Protocol

A drop of 8CB (4-n-octyl-4’-cyanobiphenyl) from Frinton Laboratories is deposited on an electronic microscopic grid with hexagonal holes (see Figure 1). The used grid, G2405C, has a diameter of 3.05 mm and is made of copper, supplied by Oxford Instruments. The number of holes can be varied by using different grids with different hole counts, and the defective texture, specifically the focal conic domain, evolves in a similar manner. This is the reason why we only focus on the grid presented in Figure 1. Annealing is carried out using an oven designed by Linkam (model LTS120) to obtain a uniform film, the profile of which is shown in Figure 1a below. In this geometry, homeotropic anchoring is achieved around the entire free-suspended film boundary, both on the top and the bottom surfaces. The smectic layers tend to remain equidistant, but due to the shape of the film, defects appear in the bisector plane of the sample. In this geometry, focal conic domains help maintain the smectic periodicity. The shape of the smectic sample is shown in Figure 1. Near the center of the grid, the film is thinner compared to the periphery. The FCDs form within a wedge-shaped meniscus. Their appearances and eccentricities are determined by the dihedral angle ω of the meniscus [19], which creates a tilt grain boundary in the smectic phase, as illustrated in Figure 1. The eccentricities vary depending on the location, approaching zero near the flat region of the meniscus. Additionally, the eccentricities are temperature-dependent, as temperature influences the surface tension, which in turn affects the geometry of the meniscus. The FCDs images were taken at the center, where the thickness is significantly reduced. However, even in the thinnest region, the sample remains sufficiently thick to allow the observation of focal conics that are as large as possible.

**Figure 2:**
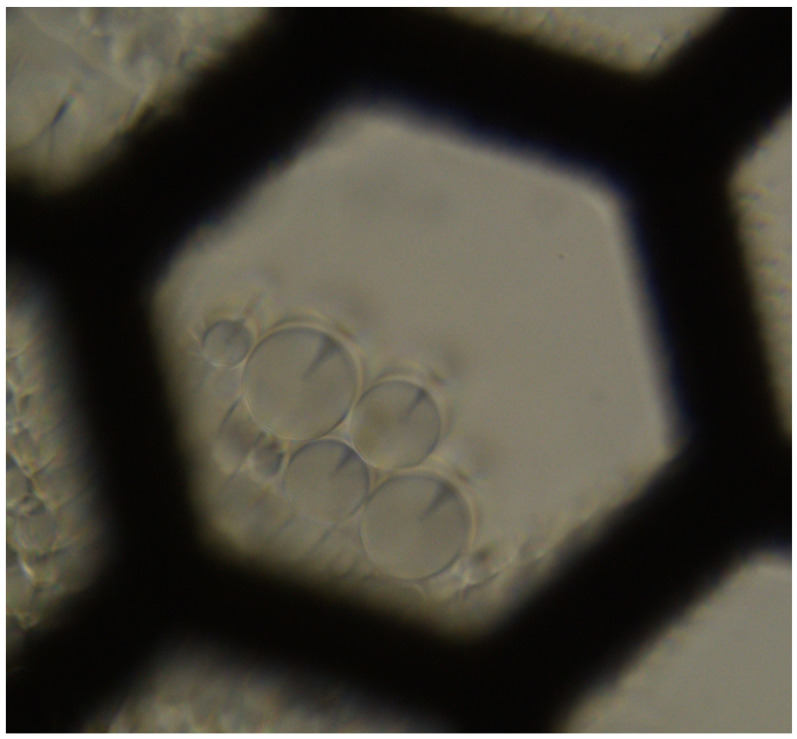
Grid with a 8CB drop observed under an optical microscope, using a crossed polarizer-analyzer setup aligned with the edges of the image. View of a hexagon-shaped hole with a side length of 175 µm.

**Figure 3:**
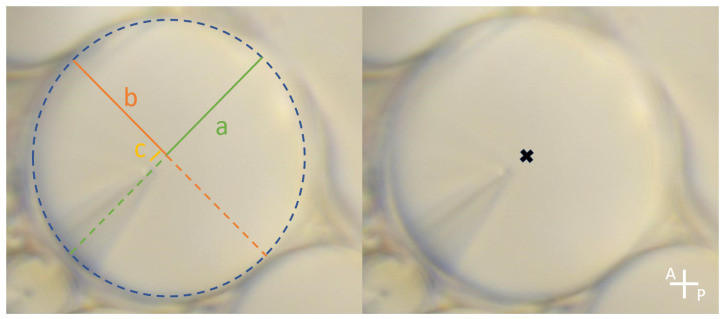
Illustration of the measurements performed on the focal conics. **Left**: Dashed line, ellipse outline; green, semi-major axis of the ellipse a; orange, semi-minor axis of the ellipse b; yellow, distance c between the ellipse center and the physical focus. **Right**: the ellipse center materialized in black has been superimposed on the experimental focal conic shapes to better appreciate the small c value and therefore the small eccentricity value.

The sample was deposited in a heating system which allows a temperature increase with a step of 0.01 K/min. Temperature has been very slowly varied with an accuracy of 0.002 K. Observations were realized with an optical polarized light microscope (Olympus BX60, Olympus Optical Co., LTD., Tokyo, Japan) using transmission. The sample was installed between optical crossed polarizers on the optical path to better view singular lines, which are in this case ellipses and confocal hyperbolas, as shown in Figure 2. The sample was initially at room temperature (∼293 K) in its solid state. Then, temperature was increased in order to reach the smectic A phase. At this stage, focal conics appear. Then, temperature continued to rise at a step of 0.01 K/min until the nematic phase. This is the instant where photographs of the sample were made, typically every tens of a second, to see how the focal conics evolve. Once the nematic phase was reached, we cooled the sample to the smectic A phase and heated it once again for other observations. Once the observation was completed, measurements were taken on each image using ImageJ Software. For each photo, we fitted an elliptical shape drawn with a dashed line, superimposed on the ellipse of the experimental focal conic shape. The software then directly provided the values of the minor 2b and 2a major axes of the ellipses, which are necessary to calculate their eccentricities $e=\frac{c}{a}=\frac{\sqrt{a^{2}-b^{2}}}{a}$. Figure 3 shows a focal conic with the added ellipse outline, which is nearly circular in this case, as well as the axes and the distance between the center and one of the two foci.

## 3. Observations

**Figure 4:**
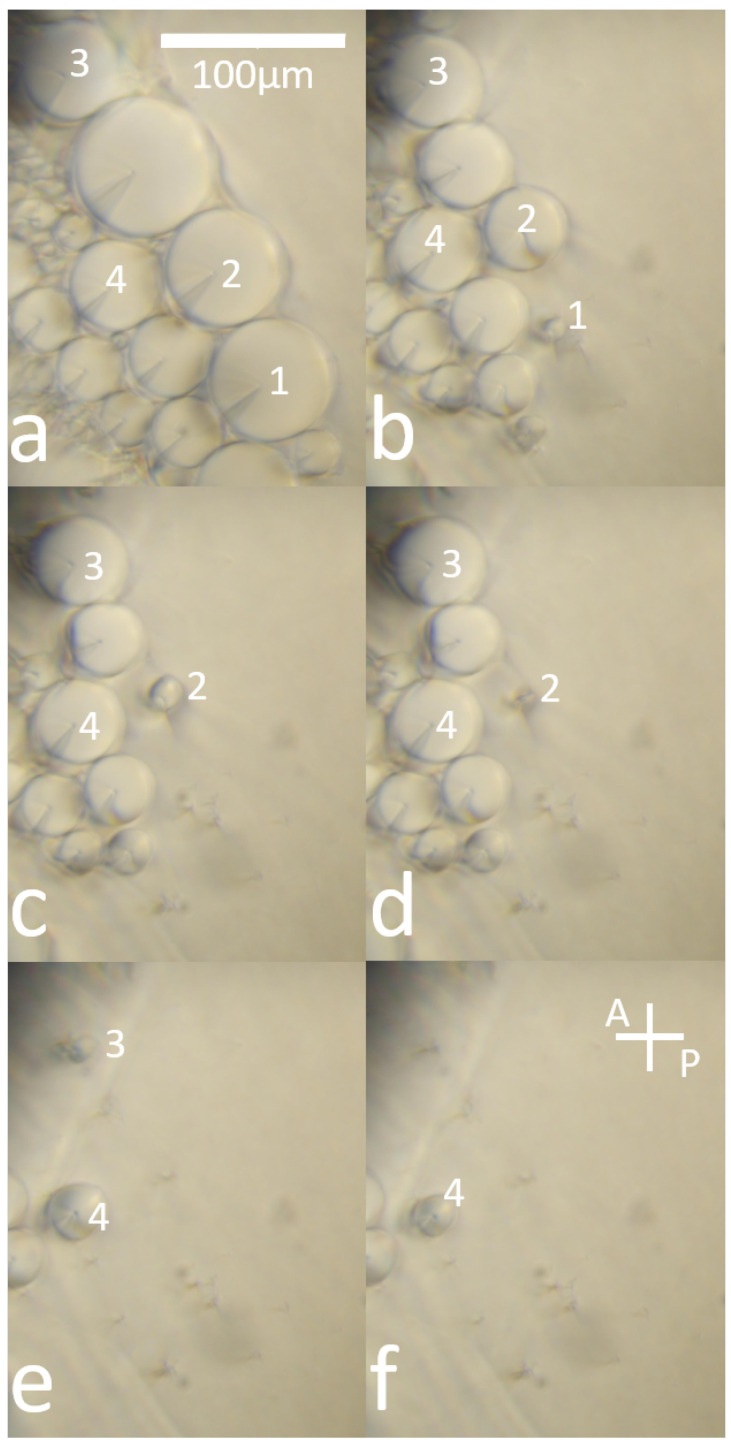
Evolution of four focal conics domain with a 0.01 K increment: (**a**) 306.211 K; (**b**) 306.231 K; (**c**) 306.234 K; (**d**) 306.234 K; (**e**) 306.239 K; (**f**) 306.242 K. Other focal conics were not used because they seem to interact all together, leading to impossible measurements. Observations were made under optical crossed polarizers. Bar = 100 µm.

**Figure 5:**
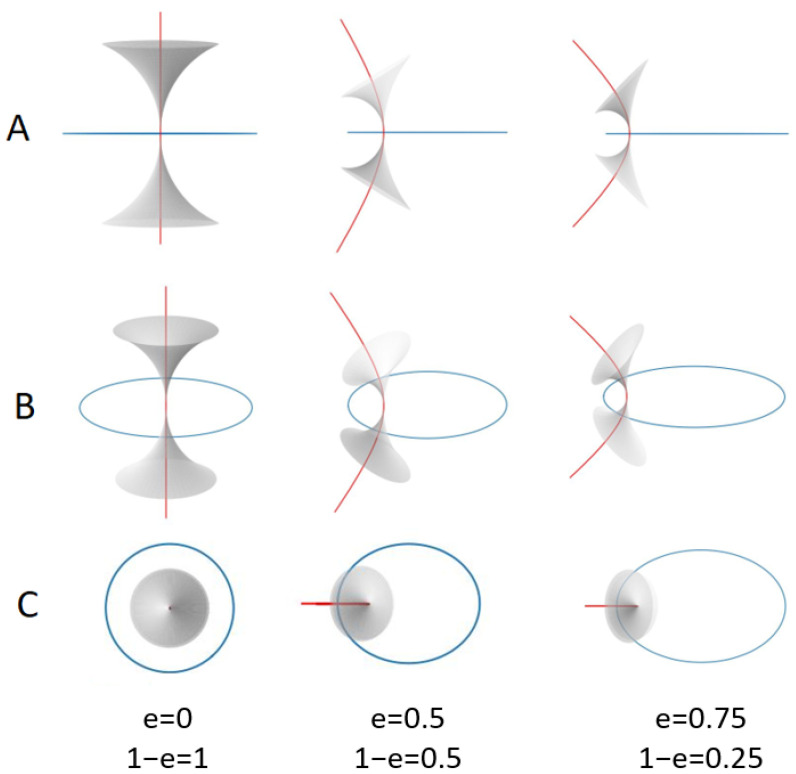
Schematic representation of the structure of a layer associated with a focal conic domain; three different eccentricity values have been used to make the simulation (e=0,0.5,0.75) from the left side to the right side. Three different views are also reported: (**A**) side view; (**B**) view at a 30° angle compared to previous figures; (**C**) top view. The hyperbolas are show in red, in blue the ellipses, and in gray a molecular smectic layer, taking the shape of a Dupin cyclide of type three. The figures have been made with Python.

Figure 4 shows the evolution of four different focal conics as the eccentricity increases, followed by their disappearance as it approaches the SmA-N phase transition. It can be compared with Figure 5A,B, which represents the shape of a focal conic with different ellipse eccentricity values. Figure 5C, especially, allows us to observe the conic from above, that is, from the same angle as the photographs in Figure 4. In fact, as already stated in [3], around focal conics, smectic layers take the shape of Dupin cyclides. For the sake of clarity, only smectic layers of type 3 are represented (see references [4] and Figure 4 of [20]).

Several observations can be made from these images.

- The ellipse has an almost circular shape when the latter is isolated from other focal conics (see Figure 4a); the shape is well-defined and can therefore be precisely measured. With an elliptical shape, the semi-minor axis *a* and the semi-major axis *b* can be obtained with ImageJ Software, see Figure 3 for an example. The apex of the hyperbola is also visible in this picture, so that we can check our estimation of the ellipse size with the measured value of the focus distance *c*, the measured value being given by $c=\sqrt{a^{2}-b^{2}}$. To estimate the errors, a series of 20 manual measurements was performed on the same focal conic. The uncertainty was determined by the deviations between the measurements.
- When temperature very slowly increases, the eccentricity tends towards 1; the disappearance of the ellipse (about 1 degree before the SmA-N transition) prevents measuring a value greater than 0.95. See, for instance, ellipse number 2 in Figure 4c or ellipse number 4 in Figure 4f.
- Each focal conic completely disappears and seems to, under the observation conditions used, leave no visible trace.
- The increase in eccentricity is a very rapid phenomenon, of the order of 0.02 s at the most.

## 4. Model

In order to model the observed phenomenon, we decided to represent the eccentricity of the ellipse with respect to temperature. Our model is characterized by the following Equation (1):

(1) $$1-e=A{\left(1-\frac{T}{T_{v}}\right)}^{\beta }$$

Here, *e* is the eccentricity, *T* the temperature. $T_{v}$ represents the temperature where the focal conics vanish, which is experimentally observed. *A* and β are two dimensionless parameters which we have to adjust on our data. We chose 1−e instead of e to obtain a convergent curve. This equation is valid when T→Tv.

β is a critical-point exponent defined by Equation (2):

(2) $$\begin{array}{c} \beta \equiv \lim_{\tau^{-}\to 0}\frac{\log(1-e)}{\log(-\tau )} \end{array}$$

where $\tau =\frac{T-T_{v}}{T_{v}}$ [21].

To determine the best values for *A* and β, we calculate the mean square deviation using the Equation (3):

(3) $$\begin{array}{cc} \Delta (\%) & =100*\sqrt{\sum_{i=1}^{n}\frac{{\left({\left(1-e\right)}_{i,calc}-{\left(1-e\right)}_{i,exp}\right)}^{2}}{n}}\\ \; & =100*\sqrt{\sum_{i=1}^{n}\frac{{\left(e_{i,exp}-e_{i,calc}\right)}^{2}}{n}} \end{array}$$

The parameter n is the total number of experimental points. The values for *A* and β which describe the focal conic vanishing are those which minimize Equation (3).

**Figure 6:**
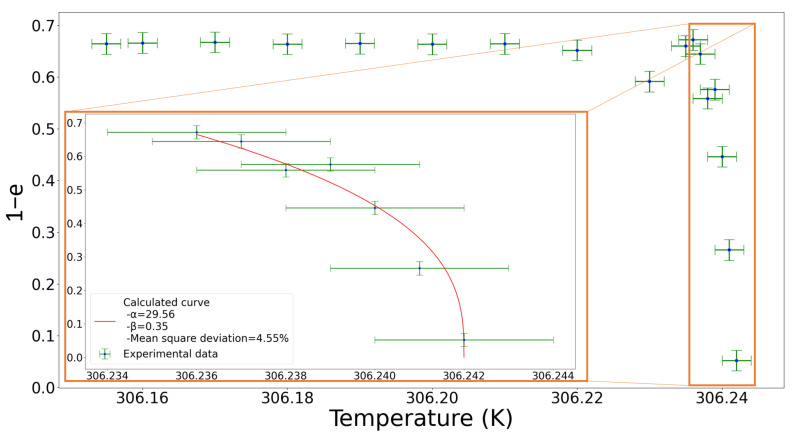
Representation of eccentricity as a function of temperature. Curves obtained for an isolated focal conic. Red curve, calculated data from the model; orange rectangle, zoom on the experimental data as the eccentricity increases.

The following graphs in Figure 6 represent the curves obtained using Equation (1), compared with the experimental measurements where the parameters *A* and β have been adjusted. The root mean square deviations range between 9.7% and 4%. The β value is estimated to be 0.34±0.02. It therefore seems that the β coefficient value is consistent with minimizing the root mean square deviation, which suggests that it should be extendable and not restricted to each individual focal conic.

## 5. Discussion

The comparison between experimental data and fit shows a good agreement during subsequent disappearance of the focal conics. Equation (1) accounts for the shape of the phenomenon with an average deviation of less than 10%. However, it accurately describes the behavior of focal conics when they disappear but does not describe their overall shape beforehand. Indeed, before fading away, focal conics remain generally stable with nearly zero eccentricity, with the only observed changes being due to interactions with other structures when they are particularly close. The eccentricity of an isolated focal conic remains near zero, and suddenly increases to one just before the SmA-N phase transition. A special point to note concerns the disappearance temperature $T_{v}$: its value is not universal but seems to depend on each of the focal conics studied. However, it is noteworthy that the smallest conics are the first to disappear. Indeed, the pearl necklace structure, composed of the smallest conics, fades first, followed by the largest conics by increasing size. It therefore appears that there is a correlation between the size of a conic and the temperature at which the eccentricity increases and then collapses. The model conducted here describes the geometric evolution of the focal conic shape with a simple mathematical equation as it approaches the SmA-N phase transition. However, this study does not fully explain the physics behind the phenomenon. Further research on the topological aspect of the liquid crystal will be necessary.

## 6. Conclusions

The model presented describes with satisfactory precision the behavior of focal conics as the smectic A to nematic transition is approached. Although this phenomenon occurs very rapidly, it was possible to observe it accurately by very gradually changing the temperature. This enables a mathematical description that reveals the exponent β, which appears to have the characteristics of a critical exponent, with its value estimated to be around 0.35. This parameter is expected to be universal for this type of second-order phase transition. Furthermore, reproductibility of this kind of experiment has been observed in a great number of samples (see Figure S1 in Supplemental Materials for an illustration). Future perspectives based on this work will consist of creating new thinner smectic A liquid crystal free-standing films in order to better understand the link between topological defects themselves.

## Data Availability

The original contributions presented in this study are included in the article/.supplementary material. Further inquiries can be directed to the corresponding author(s).

## Supplementary Materials

The following are available online at https://www.mdpi.com/article/10.3390/ma18030711/s1, Figure S1: Number of occurrences per β value.

## Abbreviations

The following abbreviations are used in this manuscript:

FCD	Focal Conic Domain

## References

[1] Frank F.C.I. (1958). Liquid crystals. On the theory of liquid crystals. Discuss. Faraday Soc..

[2] Grandjean F., Friedel G. (1910). Observations géométriques sur les liquides à coniques focales. Bull. De Minéralogie.

[3] Kleman M., Lavrentovich O.D. (2003). Soft Matter Physics: An Introduction.

[4] Kleman M., Lavrentovich O.D. (2009). Liquids with conics. Liq. Cryst..

[5] Nastishin Y.A., Meyer C. (2023). Imperfect defects in smectics A. Liq. Cryst. Rev..

[6] Rosenblatt C.S., Pindak R., Clark N.A., Meyer R.B. (1977). The parabolic focal conic: A new smectic a defect. J. Phys..

[7] Williams C. (1978). DÉFAUTS LINÉAIRES DES MÉSOPHASES SMECTIQUES A. J. Phys. Colloq..

[8] Williams P.C.E., Kleman M. (1976). Sur passociation des lignes de dislocations vis et des coniques focales dans les smectiques A. Philos. Mag..

[9] Meyer C., Nastishin Y., Kleman M. (2010). Helical defects in smectic-*A* and smectic-*A*^*^ phases. Phys. Rev. E.

[10] Meyer C., Rabette C., Gisse P., Antonova K., Dozov I. (2016). DH* in chiral smectics under electric field. Eur. Phys. J. E.

[11] Kamien R.D., Nastishin Y., Pansu B. (2023). Geometry of focal conics in sessile cholesteric droplets. Proc. Natl. Acad. Sci. USA.

[12] Gim M.J., Beller D.A., Yoon D.K. (2017). Morphogenesis of liquid crystal topological defects during the nematic-smectic A phase transition. Nat. Commun..

[13] Lochon F. (2022). Propriétés Optiques de Nanoparticules Plasmoniques et de Cristaux Liquides pour Applications aux Vitrages Intelligents. Ph.D. Thesis.

[14] Boniello G., Vilchez V., Garre E., Mondiot F. (2021). Making Smectic Defect Patterns Electrically Reversible and Dynamically Tunable Using In Situ Polymer-Templated Nematic Liquid Crystals. Macromol. Rapid Commun..

[15] Mahyaoui C.N., Davidson P., Meyer C., Dozov I. (2024). Polymerisation of twist-bend nematic textures for electro-optical applications. Soft Matter.

[16] Luo D., Wu J., Guo Z., Xia J., Hu W. (2024). Generation and regularization of zigzag focal conic domains guided by thermodynamic-driven topological defect evolution. Giant.

[17] Yoon D., Choi M., Kim Y.H., Kim M., Lavrentovich O., Jung H.T. (2007). Internal structure visualization and lithographic use of periodic toroidal holes in liquid crystals. Nat. Mater..

[18] Meyer C., Kleman M. (2005). How Do Defects Transform at the Smectic A-Nematic Phase Transition?. Mol. Cryst. Liq. Cryst..

[19] Kléman M., Lavrentovich O. (2000). Grain boundaries and the law of corresponding cones in smectics. Eur. Phys. J. E.

[20] Meyer C., Le Cunff L., Malika B., Foyart G. (2009). Focal Conic Stacking in Smectic A Liquid Crystals: Smectic Flower and Apollonius Tiling. Materials.

[21] Stanley H. (1987). Introduction to Phase Transitions and Critical Phenomena.

