# Supplementary material for: Evolution of Focal Conic Domains in SmA-N Phase Transition
Source: Materials (Basel). 2025 Feb 6;18(3):711. doi: 10.3390/ma18030711 (PMC11819984; doi:10.3390/ma18030711)
Supplement: Supplementary file 1 [file materials-18-00711-s001.zip › materials-3405106-supplementary.pdf]

Supplemental material of

Evolution of Focal Conic Domains in SmA - N phase transition

This diagram shows the number of occurrences for the different values of the β parameter calculated according to the described model.

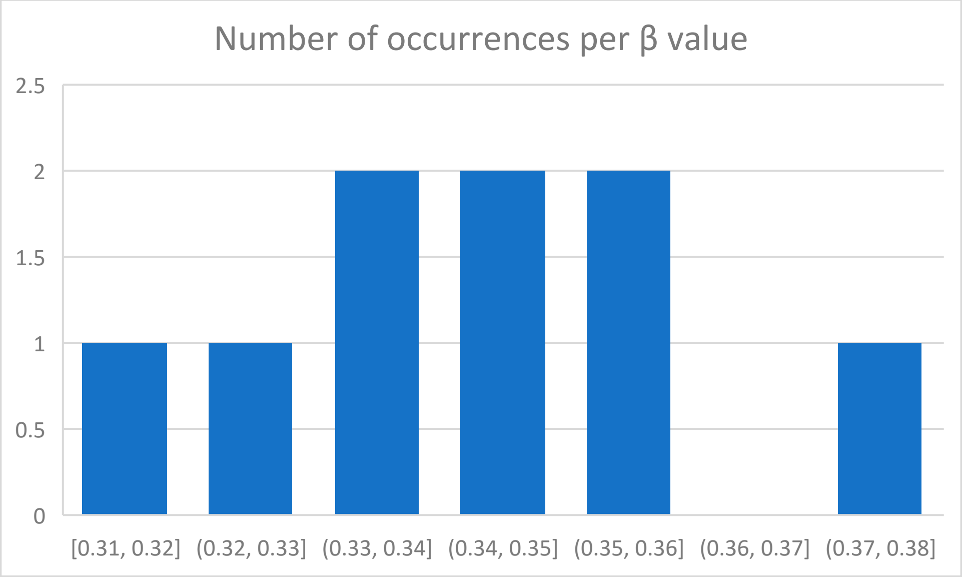

Figure S1: Number of occurrences per β value
